# Detection of a Specific Biomarker for Epstein-Barr Virus Using a Polymer-Based Genosensor

**DOI:** 10.3390/ijms15059051

**Published:** 2014-05-21

**Authors:** Renata P. A. Balvedi, Ana C. H. Castro, João M. Madurro, Ana G. Brito-Madurro

**Affiliations:** 1 Institute of Genetics and Biochemistry, Federal University of Uberlândia, Uberlândia 38400-902, Brazil; E-Mails: renataalvesbalvedi@hotmail.com (R.P.A.B.); tininha112@yahoo.com.br (A.C.H.C.); 2 Institute of Chemistry, Federal University of Uberlândia, Uberlândia 38400-902, Brazil; E-Mail: jmadurro@ufu.br

**Keywords:** Epstein-Barr virus, electrochemical detection, genosensor, hybridization, polymer

## Abstract

This paper describes methodology for direct and indirect detections of a specific oligonucleotide for Epstein-Barr virus (EBV) using electrochemical techniques. The sequence of oligonucleotide probe (EBV1) revealed a high sequence identity (100%) with the EBV genome. For the development of the genosensor, EBV1 was grafted to the platform sensitized with poly(4-aminothiophenol). After that, the hybridization reaction was carried out with the complementary target (EBV2) on the modified electrode surface using ethidium bromide as DNA intercalator. The oxidation peak currents of ethidium bromide increased linearly with the values of the concentration of the complementary sequences in the range from 3.78 to 756 μmol·L^−1^. In nonstringent experimental conditions, this genosensor can detect 17.32 nmol·L^−1^ (three independent experiments) of oligonucleotide target, discriminating between complementary and non-complementary oligonucleotides, as well as differentiating one-base mismatch, as required for detection of genetic diseases caused by point mutations. The biosensor also displayed high specificity to the EBV target with elimination of interference from mix (alanine, glucose, uric acid, ascorbic acid, bovine serum albumin (BSA), glutamate and glycine) and good stability (120 days). In addition, it was possible to observe differences between hybridized and non-hybridized surfaces through atomic force microscopy.

## 1. Introduction

Epstein-Barr virus (EBV) is a DNA virus of the γ-herpes subfamily. It infects around 90% of the world’s population and may be asymptomatic during the life of the host. Its latent state could be regarded as the causative agent of infectious mononucleosis and has been associated with several malignancies including Burkitt’s lymphoma, oral and gastric carcinomas (particularly nasopharyngeal carcinoma), Hodgkin’s disease, lymphoproliferative disorders in immunodeficient individuals, such as post-transplant lymphoproliferative diseases and a subset of T and NK cell lymphomas [1–5].

EBV was discovered 40 years ago from examining electron micrographs of cells cultured from Burkitt’s lymphoma that indicated a viral etiology and became the first of an unexpectedly wide range of associations discovered between this virus and tumors [6]. It is currently being correlated to autoimmune diseases such as multiple sclerosis, systemic lupus erythematosus, rheumatoid arthritis and primary Sjögren’s syndrome; these are complex disorders with a genetic background and the involvement of environmental factors, including viruses [7,8].

Currently, the detection of the virus can be made by means of several techniques, such as PCR (polymerase chain reaction), *in situ* hybridization, immunohistochemistry and electron microscopic immunocytochemistry. These techniques have a high cost, demanding skilled labor and time-consuming analysis [9–12]. Other recent technologies, such as the development of biosensors, are emerging and may revolutionize the disease diagnosis [13]. Generally, electrochemical biosensors have high sensitivity and low-cost, having superior properties when compared to alternative analytical techniques [14–16].

Electrochemical DNA sensing is a promising technique of nucleic acid analysis because of its fast response time, high sensitivity and low cost. This technique employs immobilized DNA sequences on the sensor surface as recognition element and sequence-specific hybridization can be monitored and analyzed [17,18]. The signal of the hybridization can be detected directly, produced by nucleotide oxidation of the DNA probe (label-free detection) [19,20] or indirectly, using an indicator of the hybridization process [21,22].

The use of conducting polymeric films can improve the interaction of the electrode with the biomolecular probe by modification of electrode surfaces, in addition to protecting the electrode against adsorption of nonspecific analytes [23–27].

Our research group has reported modifications of electrode surfaces with functionalized polymers, derived from aminophenols [28–31], tyramine [32], hydroxybenzoic acid [33], hidroxyphenylacetic acid [34] and methoxyphenethylamine [27], as well as application of the modified electrodes in the immobilization and detection of biomolecules [35–37].

The success of biomolecules immobilization onto these polymeric matrices is due to the presence of functional groups (amino, hydroxyl and/or carboxylic acid), facilitating electropolymerization and increasing the retention of biomolecules [31].

This work describes a genosensor based on a platform sensitized with poly(4-aminothiophenol)/specific single-strand DNA for EBV. The linear response range, sensitivity, selectivity, repeatability and stability were investigated.

## 2. Results and Discussion

### 2.1. Electrochemical Behavior of 4-Aminothiophenol (4-ATP)

**Figure 1.**
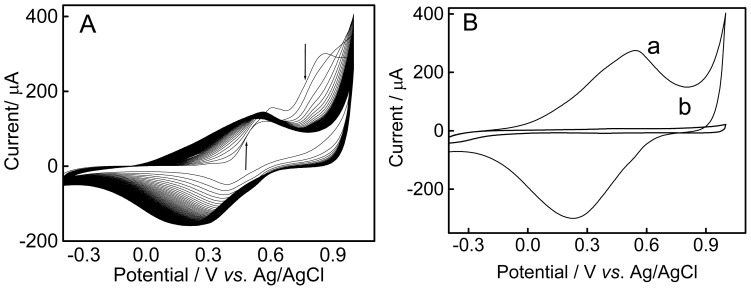
(**A**) Cyclic voltammogram in 4-aminotiophenol (4-ATP) solution (15 mmol·L^−1^) onto graphite electrode. Supporting electrolyte: H_2_SO_4_ 0.5 mol·L^−1^; Scan rate 50 mV·s^−1^; 100 scans. The arrows indicate the influence of the current response with the increasing of the number of scans; and (**B**) Cyclic voltammograms of bare graphite electrode (a) and graphite electrode functionalized with poly(4-ATP) (b). Supporting electrolyte: H_2_SO_4_ 0.5 mol·L^−1^; Scan rate: 50 mV·s^−1^.

The 4-aminotiophenol (4-ATP) electropolymerization was carried out through potential scanning (Figure 1A). The electrochemical characterization was carried out in H_2_SO_4_ solution (Figure 1B).

Figure 1A shows a gradual increase in current response between 0.0 and +0.7 V, where the two peaks of oxidation and reduction are observed, indicating the formation of electroactive material.

A decrease in the oxidation peak from +0.7 to +1.0 V is attributed to oxidation of the monomer (Figure 1A). The modification of the graphite electrode surface was confirmed in aqueous H_2_SO_4_ 0.5 mol·L^−1^ (Figure 1B).

### 2.2. Electrochemical Characterization of the Modified Electrodes

**Figure 2.**
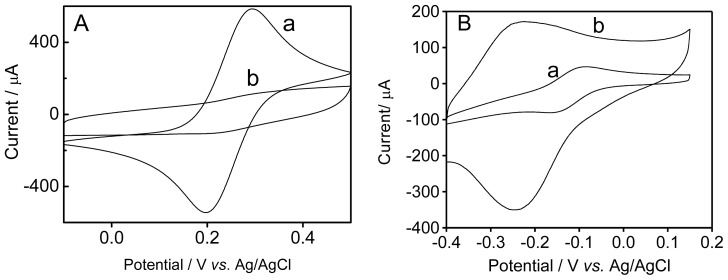
Cyclic voltammograms of bare graphite and graphite electrode modified with poly(4-ATP): (**A**) in aqueous solution containing K_4_Fe(CN)_6_/K_3_Fe(CN)_6_ (5.00 mmol·L^−1^) and KCl (0.10 mol·L^−1^); (**B**) in aqueous solution containing Ru(NH_3_)_6_Cl_2_ (5.00 mmol·L^−1^) and KCl (0.10 mol·L^−1^). Bare graphite electrode (a) and modified graphite electrode with 4-ATP (b) Scan rate: 50 mV·s^−1^.

The modified graphite electrodes were studied in aqueous solution containing [Fe(CN)_6_]^4−^/[Fe(CN)_6_]^3−^ or Ru(NH_3_)_6_^2+^ (Figure 2).

In aqueous solution containing [Fe(CN)_6_]^4−^/[Fe(CN)_6_]^3−^, the current of the modified electrode decreases, as well as a larger separation of the peak potentials is observed, denoting difficulty and, eventually, hindrance of the electron transfer to the [Fe(CN)_6_]^4−^/[Fe(CN)_6_]^3−^ system. Such behavior is also seen in other polymer layers [38,39]. The current response of the electrode modified with poly(4-ATP) is higher in presence of Ru(NH_3_)_6_^2+^, when compared with bare graphite electrode.

This model of electrostatic interaction promotes the transport of the ruthenium complex to the electrode surface, resulting in the increase of the peak current. This may be emphasized when the behavior of poly(4-ATP) towards the redox couple potassium ferrocyanide/potassium ferricyanide is evaluated, being obtained opposite results. These results indicate that the polymer has an anionic nature.

Sonmez *et al*. [40] described similar studies using hexaammineruthenium (III) chloride and sulfonated polyelectrolyte as dopant. Cation exchange properties were observed with hexaammineruthenium (III) chloride as an active electrolyte. An increase in the amplitude of the current signal was observed when compared to non-modified electrodes.

### 2.3. Immobilization and Hybridization

The electrochemical oxidation of natural and synthetic nucleic acids has also been widely studied on pyrolytic graphite electrodes, spectroscopic graphite impregnated with paraffin and glassy carbon [41].

**Figure 3.**
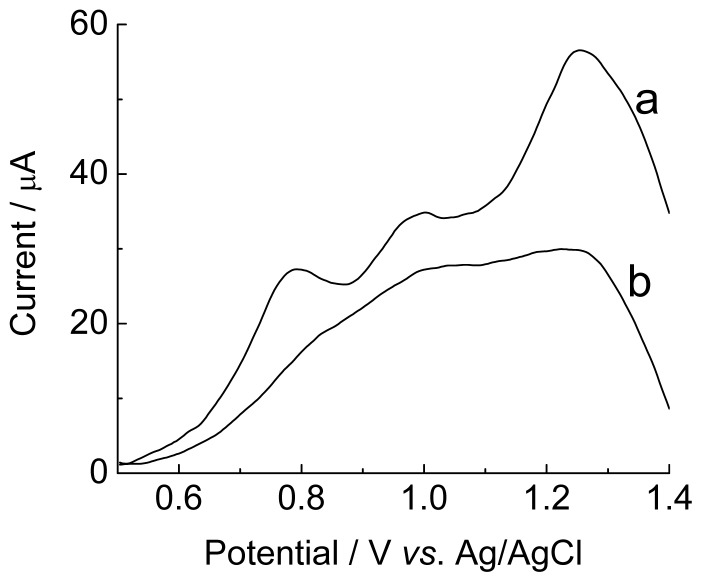
Differential pulse voltammograms of graphite electrode modified with poly(4-ATP) prepared in pH 0.5 (baseline-corrected), 100 scans, containing [EBV1, (oligonucleotide probe) 126 μmol·L^−1^]: before hybridization (a) and after 20 min of incubation with complementary target (EBV2, 378 μmol·L^−1^) (b). Electrolyte: phosphate buffer (0.10 mol·L^−1^), pH 7.4. Modulation amplitude: 0.05 mV. Pulse interval: 0.2 s. Scan rate 5 mV·s^−1^.

Figure 3 shows the response of the immobilization of the EBV1 probe and the hybridization by direct detection with the complementary target EBV1:EBV2 onto modified graphite electrode. The anchor of the DNA onto the surface of the modified electrode is not well known but, in agreement with literature [42], the presence of amine groups favors the formation of covalent binding with oligonucleotides by linkages carboxamide or phosphoramidate.

The potential peaks at +0.95 and +1.22 V are attributed to guanosine and adenosine, respectively. These peaks decreased after 20 min at 57 °C, in agreement with Oliveira-Brett *et al.* [43], who reported that hydrogen bonds are formed between complementary sequences during the hybridization of oligonucleotides, leading to a duplex, inside of which it is more difficult to oxidize the bases, decreasing the peak current of the guanosine and adenosine, after the hybridization.

Another reason for the higher current values obtained for single-stranded DNA is that the latter presents higher proximity and a higher degree of adsorption onto the electrode surface, due to its higher conformational flexibility, facilitating the charge transfer between the nitrogenous bases and the electrode [42–44].

Ethidium bromide [(3,8-diamino-5-ethyl-6-phenyl phenatridinium bromide)] is one of the best known intercalating agents, first synthesized in 1952 by Watkins and Woolfe [45]. This intercalator is largely used to visualize the nucleic acids through agarose gel electrophoresis, due to formation of a fluorescent complex. Ethidium bromide does not require changes on the DNA structure, it is a cheap product, the system can be easily constructed and the intercalation is immediate [46].

Planar aromatic organic compounds, which also include ethidium bromide, are redox-active markers used in DNA hybridization biosensors [47]. Ethidium bromide is electroactive and its electrochemical behavior was studied in several electrodes (glassy carbon [48], graphite [49], boron-doped diamond [50] and carbon nanotube [51]). Differential pulse voltammetry studies of ethidium bromide on graphite electrode modified with poly(4-aminotiophenol) were carried out by our group; this intercalator was observed to be electroactive in this modified electrode, presenting a oxidation peak in +0.75 V *vs.* Ag/AgCl.

**Figure 4.**
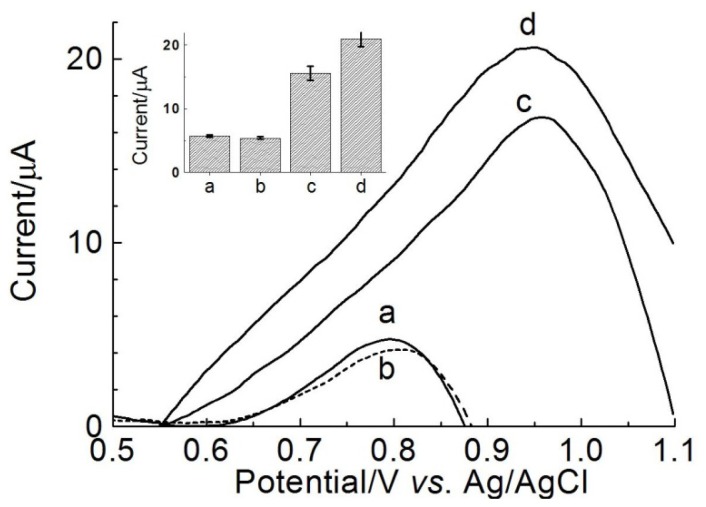
Differential pulse voltammograms of ethidium bromide (1 × 10^−6^ mol·L^−1^) onto graphite electrode modified with poly(4-ATP) prepared in pH 0.5 (baseline-corrected), 100 scans, containing EBV1/probe (126 μmol·L^−1^) before hybridization (a) and after hybridization with: non-complementary target (189 μmol·L^−1^) (b); oligonucleotide containing one-base mismatch EBV2Mis1 (378 μmol·L^−1^) (c); and complementary target (EBV2, 378 μmol·L^−1^) (d). Electrolyte: phosphate buffer (0.10 mol·L^−1^), pH 7.4. Modulation amplitude: 25 mV. Pulse interval: 0.2 s; Scan rate 20 mV·s^−1^. Inset: Bar chart of differential pulse voltammograms responses using the oxidation signal from ethidium bromide.

Figure 4 shows differential pulse voltammograms of indirect detection of target for EBV1, using ethidium bromide as electroactive indicator, in presence of complementary and non-complementary targets, as well as oligonucleotide containing one-base mismatch.

The results showed an increase in the current signal amplitude, in presence of complementary target, indicating the accumulation of ethidium bromide on the surface of the modified electrode containing duplex (Figure 4d). This accumulation indicates occurrence of hybridization process that causes intercalation of the mediator in the double-strand DNA, where a hydrophobic aromatic molecule is attracted to hydrophobic environment of nitrogenous base pairs of DNA from the hydrophilic aqueous environment [52].

Figure 4 also showed that the amplitude of the peak oxidation current for ethidium bromide is about four times higher for the complementary target, compared to the non-complementary target (see voltammograms b and d, as well as bar chart in Figure 4), indicating that the genosensor poly(4-ATP)/EBV1 discriminates complementary target from non-complementary target.

In addition, the current peak for the complementary oligonucleotide (EBV2, Figure 4d) is about 35% higher (see bar chart, inset Figure 4), when compared to oligonucleotide containing one-base mismatch (EBV2Mis1, Figure 4c), indicating that the device has potential to differentiate mismatches, as required for detection of diseases caused by point mutations. The higher potential values observed after hybridization with the complementary target are due to the higher charge transfer resistance caused by formation of the duplex.

### 2.4. Interference Studies

Blood is a complex biological fluid formed by substances such as ascorbic acid, uric acid, urea, albumin and others that can affect the response of the biosensor [52]. In the literature, it is reported that interferences can influence the accuracy of biosensors [53–55].

**Figure 5.**
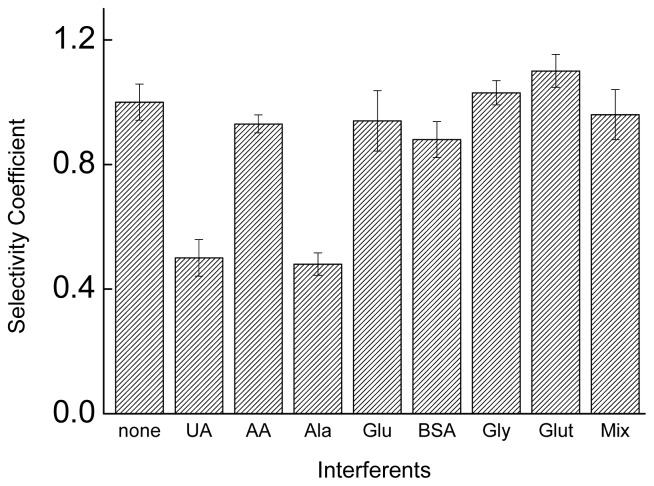
Selectivity coefficient for graphite electrode/poly(4-ATP)/EBV1 in the detection of complementary target in absence or presence of the interfering compounds: uric acid (UA) 1 mg·dL^−1^; ascorbic acid (AA) 3.6 mg·dL^−1^; glycine (Gly) 1 mmol·L^−1^; alanine (Ala) 1 mmol·L^−1^; glucose (Glu) 1 mmol·L^−1^; bovine serum albumin (BSA) 5 g·dL^−1^; glutamate (Glut) 1 mmol·L^−1^ and mixture. Electrolyte: phosphate buffer (0.10 mol·L^−1^), pH 7.4. Modulation amplitude: 25 mV. Pulse interval: 0.2 s; Scan rate 20 mV·s^−1^. Ethidium bromide (1 × 10^−6^ mol·L^−1^) was used as indicator of the hybridization.

The substances studied as potentially interfering with the electrochemical biosensor were: alanine, glucose, uric acid, ascorbic acid, BSA, glutamate, glycine and a mixture of these compounds (Figure 5).

With exception of the uric acid and alanine, the results indicate that the response of the biosensor is not significantly affected in the presence of interfering compounds, since the mixture containing all compounds studied also retained the selectivity for the detection of EBV1 (Figure 5) confirmed by mean selectivity coefficient (SC), calculated using the equation SC = *I*_c+i_/*I*_c_, where *I*_c+i_ and *I*_c_ are the bioelectrode response for the EBV2 target, in the presence and absence of each compound [53].

The selectivity studies were carried out in deionized water (pH 7). Based on the fact that the phosphate groups of the oligonucleotides are not protonated in this medium, as well as based on the pKa of the compounds studied, (alanine: 2.34 and 9.69; uric acid: 5.40 and 10.3; glycine: 2.34 and 9.60; glutamic acid: 2.16, 4.32 and 9.67; ascorbic acid: 4.17 and 11.57) [56] it is possible suggest that uric acid (positively charged) interacts with the phosphate groups, interfering with the formation of the duplex, while glutamic acid (dianion in pH 7), ascorbic acid (anion in pH 7) and BSA (isoeletric point: 4.85, negatively charged at pH 7) suffer repulsion of the phosphate and do not interfere.

The amino acids alanine and glycine show opposite behavior, although they have similar pKa and isoelectric points. The causes for this fact are not very clear, but a plausible hypothesis is that the slightly higher organic chain of alanine favors the interaction with the nitrogenous bases of the oligonucleotides, by van der Waals’ interaction, hindering the formation of the double strand. Another factor is the lower solubility of alanine in water (167.2 g·L^−1^ at 25 °C), when compared with glycine (249.9 g·L^−1^ at 25 °C), which is derived from a greater carbon chain, favoring its interaction with the nonpolar part of the oligonucleotides and competing with the formation of double-strand. The high water solubility of glucose (909 g·L^−1^, 25 °C) also suggests that the solubility is an important factor for a low interference in the formation of the duplex.

### 2.5. Calibration Curve

**Figure 6.**
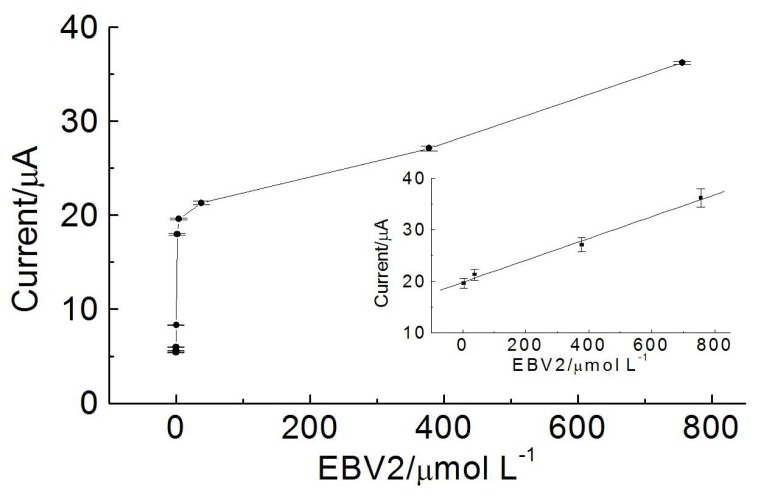
Electrochemical response for the oxidation signal of ethidium bromide (1 × 10^−6^ mol·L^−1^) obtained after the hybridization of modified electrode containing the probe EBV1 (126 μmol·L^−1^) with different concentrations of EBV2 (0, 0.0010, 0.010, 0.10, 1.89, 3.78, 37.8, 378 and 756 μmol·L^−1^). Inset shows linear range of current peak *vs.* concentration of EBV2.

Figure 6 shows the calibration curves generated using the EBV-genosensor system. The current is proportional to concentrations of EBV2 in the range from 3.78 to 756 μmol·L^−1^, with correlation coefficient of 0.998, and detection limit of 17.32 nmol·L^−1^ (three independent experiments).

### 2.6. Genosensor Stability

In order for commercialization of a biosensor to be feasible, it should have good selectivity and stability during storage to assure reproducibility of measurements. Long-term lifetime is not only beneficial to biosensor transport and storage, but it also helps decrease per measurement costs, of critical importance in pharmaceutical and industrial applications [57].

**Figure 7.**
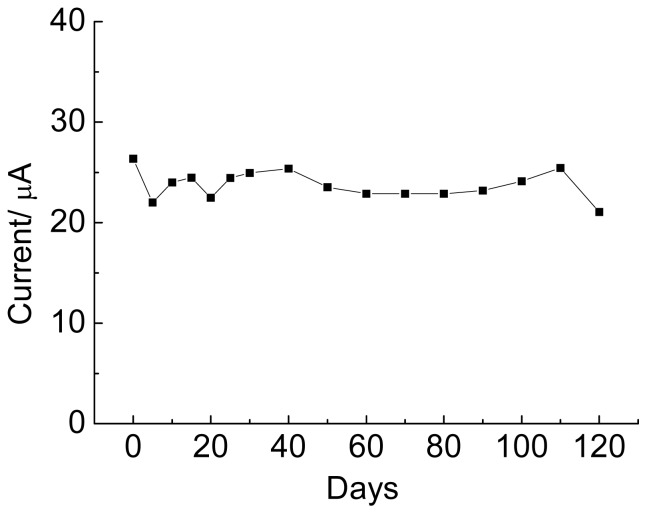
Storage stability profile of genosensor at 8 °C. The biosensors were stored in refrigerators when not in use.

The stability study of the biological sensor poly(4-ATP):EBV1 is shown in Figure 7. For this analysis, the electrodes were at a temperature of 8 °C for 120 days. During this time, assays using ethidium bromide were performed.

Figure 7 shows that the sensor response remains stable, without loss in biological activity during 120 days, indicating that the electrode modified with poly(4-aminothiophenol) contributed to this stability. Infrared spectroscopy studies of poly(4-aminothiophenol), conduced by our group, indicate that the electropolymerization occurs by formation of a ring-NH-ring bond, with the aromatic thiol being preserved in the polymer. The chemisorption of aromatic thiols at the carbon surface is known in the literature [58], and the presence of amine groups favors the formation of covalent binding with oligonucleotides, indicating that a modification of the electrode surface with poly(4-aminothiophenol) favors the stability and maintenance of biological activity of the device.

### 2.7. Morphological Characterization of the Genosensor Using Atomic Force Microscopy

**Figure 8.**
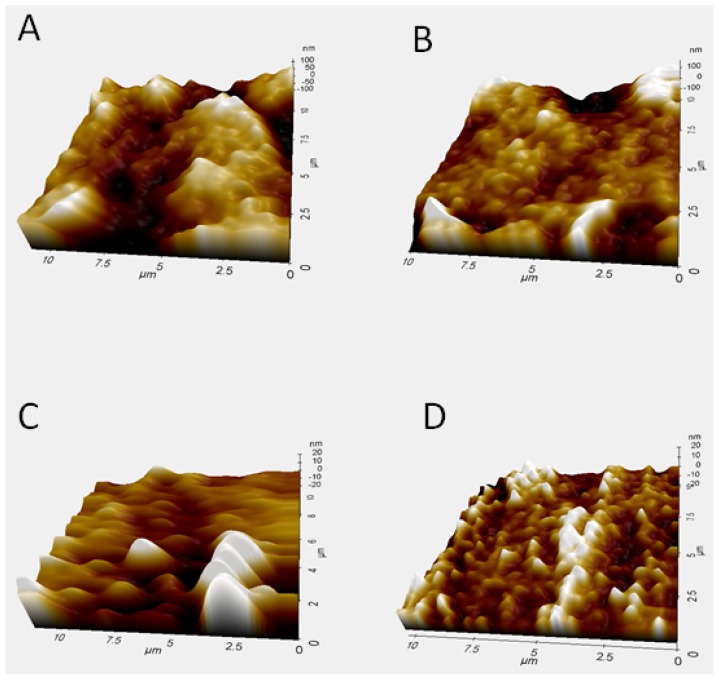
Atomic force microscopy (AFM) images of (**A**) graphite; (**B**) graphite/poly (4-ATP); (**C**) graphite/poly(4-ATP)/EBV1; and (**D**) graphite/poly(4-ATP)/EBV1:EBV2.

Analyses of the surface for bare graphite electrode and graphite modified with poly(4-ATP) in absence or presence of the complementary target, are shown in Figure 8.

Roughness values obtained by AFM were: 102.3 ± 4.5 nm (bare graphite electrode), 69.7 ± 6.2 nm [modified electrode with poly(4-ATP)], 13.2 ± 3.5 nm [modified electrode with poly(4-ATP)/EBV1] and 31.8 ± 5.2 nm [modified electrode with poly(4-ATP)/EBV1:EBV2].

As shown in Figures 8B,C, the immobilization of EBV1 produces a decrease in the height and size of the clusters when compared to modified electrode with poly(4-ATP) without biomolecules (Figure 8A), indicating that the oligonucleotide was successfully incorporated on the electrode surface. Both modified electrodes containing poly(4-ATP)/EBV1 (Figure 8C) and poly(4-ATP)/EBV1:EBV2 (Figure 8D) showed topographies with globular aspect, but the latter is less homogenous, presenting larger clusters. These modifications in the electrode surface can be justified based on the fact that double strand DNA molecules are more elongated and inflexible than single strand DNA, and can form larger structures, suggesting the occurrence of the hybridization event, in agreement with the results obtained in the electrochemical studies. It was demonstrated in the literature that DNA can penetrate conducting films of polypyrrole [59].

## 3. Experimental Section

### 3.1. Reagents

All reagents used were of analytical grade and used without further purification. Ultra-high purity water (Master System, Gehaka, Brazil) was used for the preparation of aqueous solutions. 4-Aminothiophenol (Acros Organics, Geel, Belgium) (15 mmol·L^−1^) was prepared in ethyl alcohol (PA) and H_2_SO_4_ solution (0.5 mol·L^−1^) immediately before use. Phosphate buffer solution 0.1 mol·L^−1^ was prepared at pH 7.4. All experiments were conducted at room temperature (25 ± 1 °C). The oligonucleotides were synthesized by Invitrogen Life Technologies (São Paulo, Brazil) with the following sequences: probe: (EBV1): 5′-AGGGATGCCTGGACACAAGA-3′, complementary target (EBV2): 5′-TCTTGTGTCCAGGCATCCCT-3′, non-complementary target: 5′-ACAACCCGTTGG ACTAAC-3′ and (EBV2Mis1): TCTTGTCTCCAGGCATCCCT-3′. Stock solutions of the 3.15 × 10^−4^ mmol·L^−1^ probe and 9.45 × 10^−4^ mmol·L^−1^ target oligonucleotides were prepared in SSC 6× buffer (0.9 mol·L^−1^ NaCl, 90 mmol·L^−1^ sodium citrate, pH 7.0) and stored in a freezer until use. Buffer components (CH_3_COOH and CH_3_COONa or Na_2_HPO_4_ and NaH_2_PO_4_) were purchased from Sigma-Aldrich Chemical (St. Louis, MO, USA) (ACS purity) and prepared at pH 4.5 or 7.45, respectively. All reagents were used as received. The experiments were conducted at room temperature (25 ± 1 °C).

Analysis of the nucleotide sequence (Table 1) was performed using the Blast program (Basic Local Alignment Search Tool, obtained in http://www.ncbi.nlm.nih.gov/blast).

### 3.2. Apparatus

Electrochemical polymerization and voltammetric measurements were performed using a potentiostat (CH Instruments, model 460C, Austin, TX, USA), with a graphite disk (6 mm diameter) cut from a graphite rod (99.9995%, Alfa Aesar) as working electrode. Platinum was used as counter electrode. All potentials are referred to the silver-silver chloride reference electrode (Ag/AgCl). The graphite surface, prior to electropolymerization, was mechanically polished with alumina slurry (0.3 μm diameter), ultrasonicated, washed with distilled water and dried in the air. All solutions were degassed by nitrogen bubbling. Film morphology in absence or presence of biomolecules was assessed through atomic force microscopy (AFM) (Park System, model XE-70, Suwon, Korea).

### 3.3. Electrochemical Polymerization

The monomer solutions were degassed with N_2_ prior to electropolymerization. Poly(4-aminothiophenol) films were electrodeposited onto the graphite electrodes from a solution containing 4-aminothiophenol (15 mmol·L^−1^). The electrochemical experiments were conducted at room temperature (25 ± 1 °C), 50 mV·s^−1^, −0.4 and +1.0 V, 100 scans in three-compartment cell.

### 3.4. Electrochemical Characterization

The evaluation of ion transport was carried out in K_4_Fe(CN)_6_/K_3_Fe(CN)_6_ solution (5.0 mmol·L^−1^) containing KCl 0.10 mol·L^−1^ (negative probe) or Ru(NH_3_)_6_Cl_2_ solution (5.0 mmol·L^−1^) containing KCl 0.10 mol·L^−1^ (positive probe).

### 3.5. Oligonucleotide Immobilization onto Graphite Electrode/Poly(4-ATP)

The immobilization of oligonucleotide was carried out by applying two consecutive layers of 13 μL of 3.15 × 10^−4^ mmol·L^−1^ of the probe (EBV1) on the modified electrode surface and dried at the temperature of 37 ± 1 °C. Then the electrode was immersed in 10 mL phosphate buffer with agitation (0.1 mol·L^−1^, pH 7.4) and dried in N_2_. The blocking of the binding of non-specific biomolecules on electrode surface was done with BSA 0.5% (*w*/*v*) for 3 h, then the electrode was immersed in 10 mL phosphate buffer with agitation (0.1 mol·L^−1^, pH 7.4) and dried in N_2_. After that, differential pulse voltammetry (DPV) measurements in one-compartment electrochemical cell connected to a potentiostat were obtained by using 1 mL phosphate buffer (0.1 mol·L^−1^, pH 7.4) as electrolyte to evaluate the electrochemical sign of modified electrode with the probe.

### 3.6. Investigation on the Hybridization of Oligonucleotide Immobilized onto Poly(4-Atp) Using Guanine and Adenine Monitoring or Ethidium Bromide as Redox Indicator

For the direct detection, 26 μL of target (EBV2, 378 μmol·L^−1^) were applied to the modified electrode with EBV1, 126 μmol·L^−1^. The hybridization was carried out at 57 °C for 20 min. Then the electrode was immersed in phosphate buffer (0.1 mol·L^−1^, pH 7.4) and dried in N_2_. The annealing temperature of the oligonucleotide (EBV1) was obtained by gene runner software (version 3.01, obtained in http://www.softpedia.com/get/Science-CAD/Gene-Runner.shtml).

For the indirect detection, after the immobilization of the oligonucleotide probe (EBV1), 26 μL of target (EBV2, 378 μmol·L^−1^) were applied on modified electrode. Hybridization was carried out at 57 °C for 20 min. Then the electrode was immersed in phosphate buffer (0.1 mol·L^−1^, pH 7.4) and dried in N_2_. In sequence, 18 μL of 1 μmol·L^−1^ ethidium bromide solution (3,8-diamino-5-ethyl-6-phenylphenatridinium bromide) from Merck Millipore (Darmstadt, Germany) in ultra-high purity water were applied on the electrode surface during 5 min. Ethidium bromide binds nucleic acids via intercalative mode and causes major changes to DNA. To evaluate electrochemical sign of ethidium bromide, differential pulse voltammetry measurements were conducted using phosphate buffer (0.1 mol·L^−1^, pH 7.4) as electrolyte for the evaluation of electrochemical sign of electrode modified containing EBV1:EBV2.

### 3.7. Specificity of the Biosensor

DNA hybridization is based on the ability of the probe to recognize its corresponding complementary target. To verify the specificity of the probe, 26 μL of the non-complementary target (5′-ACAACCCGTTGGACTAAC-3′, 378 μmol·L^−1^) or one-base mismatch (EBV2Mis1: TCTTGT CTCCAGGCATCCCT-3′, 189 μmol·L^−1^) were added on surface of the sensor subjected to the same process of the complementary target. The hybridization was carried out at 57 °C for 20 min. Detection of complementary target was done indirectly through the use of a mediator.

### 3.8. Analysis of Interfering Compounds

For this study, we added the complementary target concentration normally found in the blood to the solution of the interfering compounds (3.6 mg·dL^−1^ ascorbic acid, 1 mg·dL^−1^ uric acid, 1 mmol·L^−1^ alanine, 1 mmol·L^−1^ glutamate, 1 mmol·L^−1^ glucose, 1 mmol·L^−1^ glycine, 5.0 g·dL^−1^ albumine and mixture of these compounds). All experiments were conducted at room temperature (25 ± 1 °C).

### 3.9. Stability Studies

To evaluate the biosensor stability, the modified electrodes containing DNA probe (EBV1) were stored at 4 °C, protected from light and oxygen during 120 days.

### 3.10. Calibration Curve

To evaluate the sensitivity of genosensor, 26 μL of different concentrations of complementary target (EBV2), 0, 3.78, 37.8, 378 and 756 μmol·L^−1^ were added to the genosensor. Hybridization was carried out at 57 °C, for 20 min. For the detection, ethidium bromide (1 μmol·L^−1^, 18 μL) was added to the electrode surface, for 5 min.

## 4. Conclusions

The results showed that functionalized surfaces with poly(4-ATP) are interesting platforms for the development of a genosensor. The produced genosensor shows interesting properties, such as good stability, selectivity and sensibility. This is a promising technique of molecular analysis of a specific biomarker for Epstein-Barr virus. Further studies will extend the system to determination of EBV in serum, plasma, and saliva samples.

## Figures and Tables

**Table 1.** The sequence of EBV1 has identity with Epstein-Barr virus.

Organism	BLASTn			

	Query cover	E value	Max ident	Access number
Epstein-Barr virus (EBV) genome, strain B95-8	100%	0.079	100%	V01555.2
Epstein-Barr virus (B95-8 isolate)	100%	0.079	100%	K03333.1
Epstein-Barr virus (AG876 isolate)	100%	0.079	100%	K03332.1
Epstein-Barr virus, artifactual joining of B95-8	100%	0.079	100%	M80517.1

E value (Expectation value): number of different alignments with scores equivalent to or better than the score that is expected to occur in a database search by chance. The lower the E value, the more significant is the score.
